# A Case of Idiopathic Portal Vein Thrombosis in an Immunocompetent Female

**DOI:** 10.7759/cureus.18817

**Published:** 2021-10-16

**Authors:** Joseph Park, Timothy Chong, Talha A Awwal, Hafiz M Aslam, Sara L Wallach

**Affiliations:** 1 Internal Medicine, Drexel College of Medicine, Philadelphia, USA; 2 Internal Medicine, St. Francis Medical Center, Trenton, USA; 3 Hematology-Oncology, East Carolina University, Greenville, USA

**Keywords:** portal vein thrombosis, cirrhosis

## Abstract

Portal vein thrombosis (PVT) is characterized by a complete or partial occlusion of the portal vein by a thrombus. The formation of the thrombus is usually attributed to an underlying condition that is causing a hypercoagulable state, such as malignancy or cirrhosis. When these causes are ruled out, a hypercoagulable workup can reveal other underlying prothrombotic etiologies. Still, some cases of PVT occur without any definitive underlying condition, leading to the diagnosis of idiopathic PVT. This occurred in our patient, a 53-year-old female who presented with PVT but had no clear underlying condition that led to her pathology after an extensive medical investigation.

## Introduction

Portal vein thrombosis (PVT) is commonly seen in patients with liver cirrhosis, underlying malignancy, or other prothrombotic states. The pathophysiology behind PVT can be explained using Virchow’s triad. Stasis of blood flow due to liver cirrhosis, hypercoagulable state due to a myeloproliferative disorder, or vascular endothelial injury caused by a local abdominal infection can all be potential triggers for a PVT [1]. Liver cirrhosis is by far the most significant risk factor found in patients with PVT. The presence of cirrhosis increases the relative risk of developing PVT by 7 times compared to the general population [2]. However, in non-cirrhotic cases of PVT, there are other risk factors that may contribute to the development of PVT such as myeloproliferative disorder, local abdominal infections like pancreatitis, or malignant portal vein obstruction. One study reports that an underlying cause for PVT can be identified in up to 80% of cases [3].

Thus, in the evaluation of PVT, it is important to take a thorough history from the patient, as well as begin a variety of lab tests to rule out the most common factors that may be the underlying cause behind the PVT. We report a patient who was found to have portal vein thrombosis, and an extensive workup was done to find causative factors such as malignancy and other prothrombotic disorders, but none were identified.

## Case presentation

A 53-year-old female presented to the emergency department complaining of abdominal pain that started one day prior. Her past medical history was significant for hypertension and type 2 diabetes. Located in the hypogastric region, the pain woke her from sleeping at night, was constant in character, and described as six out of 10 in severity. The pain was radiating to her right lower quadrant and she did not report any alleviating or aggravating factors. Her medications included enalapril and liraglutide. Surgical history was significant for cholecystectomy and hysterectomy, both of which were over eight years ago. She denied having any family history of malignancy, bleeding issues, or thrombotic events.

Her vital signs and laboratory studies, including hematologic and metabolic panels were within normal limits with the exception of an elevated ESR of 41 mm/hr. An abdominal ultrasound showed no significant findings. A subsequent CT scan showed findings suggestive of acute colitis involving the descending and sigmoid colon. The patient was treated for colitis with antibiotics. 

**Figure 1:**
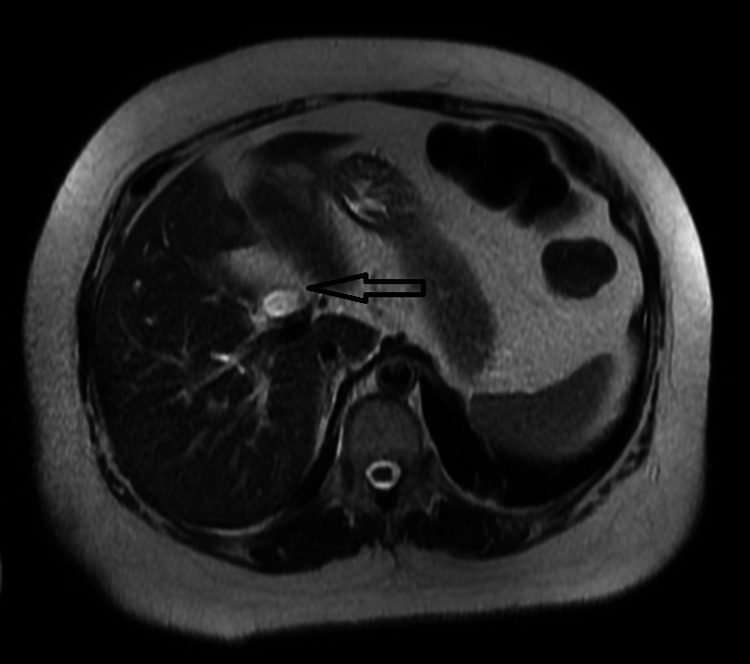
MRCP: biliary dilatation with a shouldering appearance in the ampulla region. MRCP: magnetic resonance cholangiopancreatography.

**Figure 2:**
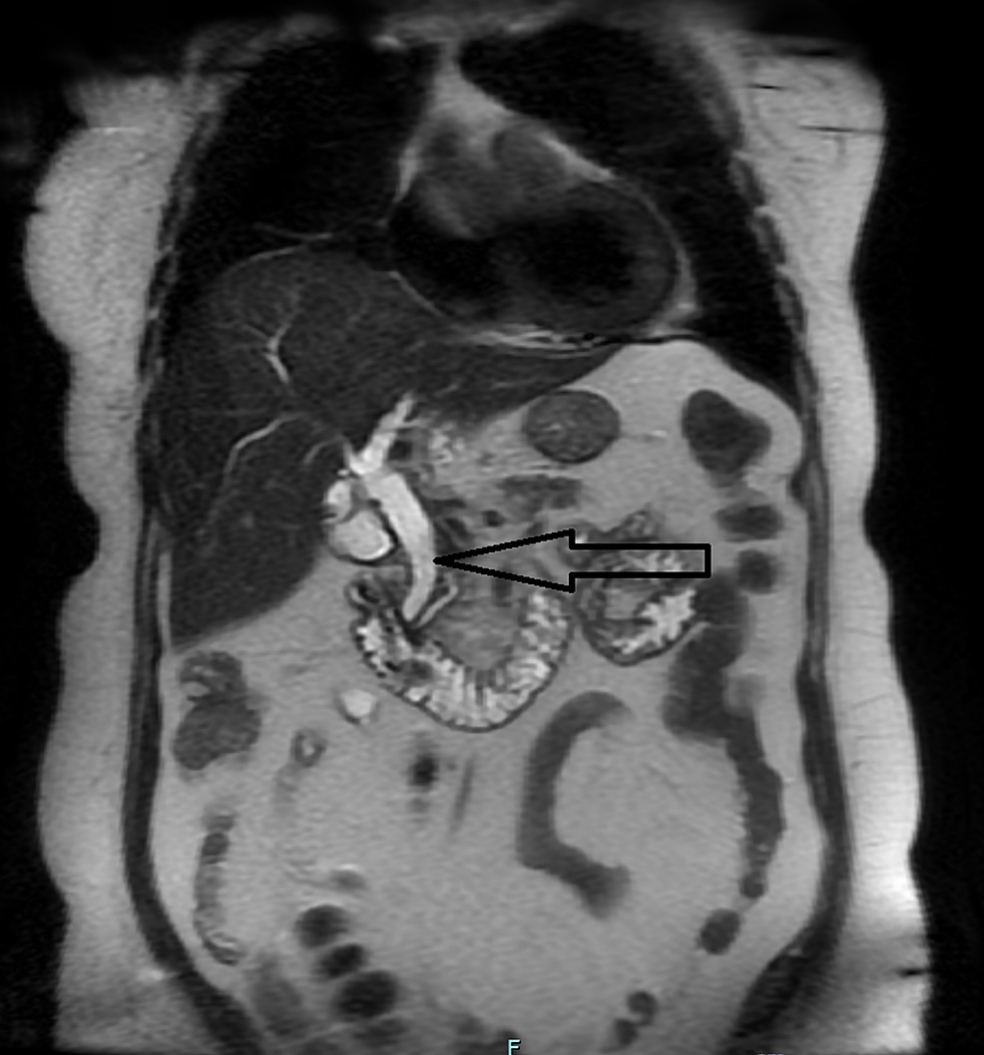
MRCP: common biliary duct dilatation. MRCP: magnetic resonance cholangiopancreatography.

Two days later, amidst no clinical improvement in the patient, the CT report was modified. The modified report was read as showing intrahepatic biliary ductal dilatation, concerning possible cholangitis or other biliary pathology. An MRCP was performed, which showed portal vein thrombosis. The MRCP (magnetic resonance cholangiopancreatography) also revealed biliary dilatation with a shouldering appearance in the ampulla region, raising concern for a possible infiltrative neoplasm. At this point, coagulation studies showed elevated levels of fibrinogen (436 mg/dL) and D-dimer (0.84 ugFEU/mL). Following this result, a hypercoagulable workup was initiated and treatment was started with heparin. An endoscopic ultrasound was unremarkable, revealing a borderline enlarged ampulla without masses. A biopsy of the region was taken which did not contain any abnormal tissue (Figures 1, 2).

The hypercoagulable workup was unremarkable. All the following laboratory results were in normal limits: antithrombin III activity, cardiolipin antibodies, factor V Leiden, protein c, protein s, phospholipid antibodies (beta-2 glycoprotein, phosphatidyl serine). She was not found to have a prothrombin gene or JAK2 (V617F) mutation. After performing an extensive hypercoagulable workup, no specific etiology for her PVT was found and she was diagnosed with idiopathic portal vein thrombosis. She was discharged shortly after in a stable condition.

## Discussion

While there is usually a cause that explains the development of PVT in a patient, some patients may have no identifying trigger or risk factor. In one study that reported findings from over 23,000 autopsies, the prevalence of PVT in the general population is around 1%. Of the patients with PVT, 14% of them did not have any major identifiable cause including malignancy, myeloproliferative disorder, or major abdominal infection/inflammatory disease [4].

PVT can be completely asymptomatic, and may only be found incidentally on imaging studies. A study on non-cirrhotic patients with PVT found that 91% of patients had a complaint of abdominal pain, and 53% of patients were found to have fever [5]. Other signs and symptoms include colicky pain associated with nonbloody diarrhea, ascites, and splenomegaly [3,6]. In severe cases, if the thrombus involves the superior mesenteric vein, it is possible to develop mesenteric ischemia and bowel infarction, which is one of the main causes of increased mortality in patients with PVT [1].

A variety of imaging modalities may be used to diagnose PVT. Because up to a third of patients with PVT may not reveal a thrombus on normal ultrasound, Doppler imaging can be used to show the absence of flow within the vessel lumen [7]. Ultrasound with Doppler imaging has been estimated to be up to 93% sensitive and specific for diagnosing PVT, although the accuracy of the imaging can be affected by individual technical skills of each ultrasonographer [8]. Abdominal CT and MRI can also be used to diagnose PVT. These methods have the advantage of high image quality regardless of variables that may affect ultrasound quality (.ie., large body habitus, bowel gas, technical skill) [3]. Subsequently, the sensitivity and specificity of MRI in diagnosing PVT has been cited to be 98% and 100%, respectively [9].

After the diagnosis of PVT has been made, if there is no clear etiology of the PVT, an extensive hypercoagulable workup should be performed. The workup involves ruling out diseases that cause prothrombotic states. Testing for these underlying causes is recommended in patients with a lifespan of greater than 6 months as they are likely to benefit from treatment. 

Prognosis of patients with PVT is good if the complication is diagnosed early and treated before intestinal infarction occurs. However, in the event of bowel infarction or multi-organ failure, hospitality mortality has been cited between 20% and 50% [10]. Treatment for PVT typically involves anticoagulation with heparin and switch to coumadin. A 2011 systematic review states that up to 83.3% of acute cases of PVT do not recanalize without the aid of anticoagulation therapy [11]. Generally, treatment should be given for six months, as recanalization has largely been found to occur in this time frame. More invasive treatments including surgical thrombectomy, thrombolysis, or transjugular intrahepatic portosystemic stent shunt (TIPS) are other alternatives to treatment. These treatments appear to be more dangerous than anticoagulation alone, although no formal comparison between these classes of treatment methods has been done [12].

Aside from the prothrombotic disorders, a number of risk factors have been described in previous literature to be associated with PVT. These include disorders associated with abdominal inflammation, such as pancreatitis, diverticulitis, inflammatory bowel disease, abdominal trauma and abdominal surgery [13].

## Conclusions

As outlined in our case description, our patient initially had findings suspicious of acute colitis, which may have put her at risk of PVT due to abdominal inflammation. While an interesting finding, this relationship is currently poorly described and there is no causal relationship between abdominal inflammation and PVT discussed in the medical literature. Thus, we are unable to elucidate if the acute colitis found on our patient’s initial CT scan was the clear underlying reason that she experienced PVT. Because a significant percentage of PVT occurs without any clear underlying cause, further investigations of risk factors (like abdominal inflammation) and their relationship with PVT should be performed so clinicians can adequately diagnose and treat patients who may suffer from this potentially fatal disease state.
